# A qualitative study of telemedicine in heart failure care

**DOI:** 10.3389/fmed.2026.1804849

**Published:** 2026-03-18

**Authors:** Valentina Micheluzzi, Chiara Idini, Giuseppe Serra, Silvia Schirru, Francesco Burrai, Eleonora Marongiu, Antonella Canu, Antonio Sircana, Pierluigi Merella, Stefano Bandino, Ferruccio Bilotta, Giovanni Maria Soro, Gavino Casu

**Affiliations:** 1 Clinical and Interventional Cardiology, Sassari University Hospital, Sassari, Italy; 2 Department of Medicine, Surgery and Pharmacy, University of Sassari, Sassari, Italy; 3 University of Sassari, Sassari, Italy

**Keywords:** digital health, heart failure, qualitative research, telemedicine, telemonitoring

## Abstract

**Background and aims:**

Heart failure is a prevalent chronic condition with a poor prognosis that impairs quality of life and generates high healthcare use. Telemedicine is increasingly used in heart failure care, but patients’ everyday experiences remain underexplored. This study aimed to explore and interpret the experiences and motivations of patients with heart failure participating in a telemedicine programme.

**Methods:**

We conducted a qualitative content analysis among patients with heart failure receiving home-based telemedicine. Semi-structured in-person interviews were audio-recorded, transcribed verbatim and analyzed inductively. The Consolidated Criteria for Reporting Qualitative Research guided conduct and reporting.

**Results:**

Data saturation was achieved after 21 interviews. Five main categories and 13 subcategories were identified: (1) benefits of telemedicine (perceived safety and reassurance, greater self-control, recommendation of telemedicine); (2) impact on daily life (time impact, practical limitations and mobility, conflicting experience); (3) relationship with healthcare professionals (presence of healthcare professionals, desire for greater contact, professional behaviors); (4) interaction with technology (unreliable devices, unreliable connectivity); and (5) family context (family support, family members’ attitudes).

**Conclusion:**

Telemedicine for patients with heart failure can enhance perceived safety and reassurance, self-management, continuity of care and treatment adherence when supported by strong patient–clinician relationships, reliable technology and alignment with daily routines and family dynamics. Future programs should be co-designed with patients and caregivers, include flexible personalized monitoring, clearly explaining how data are used and ensure two-way communication. Multicenter mixed-methods studies are needed to refine person-centered telemedicine models that best support heart failure care.

## 1 Introduction

Heart failure (HF) is a chronic, progressive syndrome characterized by structural and/or functional cardiac abnormalities accompanied by signs and symptoms of congestion. It remains a leading cause of morbidity, mortality, and healthcare expenditure worldwide (1, 2), with approximately 64 million people affected globally, and prevalence increasing with age (3, 4). Despite advances in guideline-directed therapy, prognosis is poor, with about half of patients dying within 5 years of a first diagnosis (5). Beyond its epidemiological burden, HF substantially impairs physical, psychological, and socioeconomic wellbeing, particularly in younger patients who may experience severe fatigue, dyspnea, frequent exacerbations, reduced work capacity, financial strain, and elevated anxiety and depression (6). These challenges highlight the need for coordinated, long-term, patient-centered care (7). Current HF management emphasizes multidisciplinary disease-management programs, optimization of therapy, reinforcement of self-management skills, integration of patient-reported outcomes, and supportive interventions such as psychological counseling and remote monitoring (8, 9).

Telemedicine, a core domain of digital health, encompasses remote monitoring, televisits, teleconsultation, teleassistance, tele-reporting, and other ICT-mediated interactions between patients and healthcare professionals. For chronic cardiovascular conditions, telemedicine has evolved from experimental projects to structured programs embedded in regional health systems and supported by national frameworks (10). Its deployment, accelerated by the COVID-19 pandemic, offers several advantages: reduced hospitalizations, improved functional capacity, enhanced patient satisfaction, and decreased travel, waiting times, and healthcare costs (11–13). Evidence also suggests benefits for treatment adherence, self-care behaviors, and timely therapeutic adjustments in response to early decompensation (14, 15), with ongoing trials evaluating the effectiveness and cost-effectiveness of complex digital HF platforms (16–21). The COVID-19 pandemic also acted as a catalyst for a broader reconfiguration of out-of-hospital HF care. During the pandemic, several health systems reported fewer hospitalizations for acute cardiovascular conditions, including HF, alongside disrupted ambulatory pathways and delayed presentations, raising concerns about unmet needs and adverse outcomes outside the hospital setting (22). In this context, telemedicine has been increasingly framed not only as a monitoring technology, but as a strategic instrument to redesign outpatient and home-based HF management beyond the emergency phase—supporting continuity of care, structured follow-up, and earlier clinical responses to signs of decompensation (23).

Although quantitative studies support the feasibility and clinical impact of telemedicine, its long-term success depends on understanding how patients and caregivers experience these technologies in daily life. Existing qualitative research provides insights into perceived safety, self-management, and relationships with clinicians and family, but remains limited and focused mainly on specific telemedicine components in Northern European or North American contexts (24–27). In particular, there is a gap in the literature regarding patients’ lived experience of wearable-enabled telemonitoring embedded in structured programs integrated into HF care, where remotely collected physiological data support ongoing follow-up through predefined alert thresholds and an organized clinical response pathway. This study explores the experiences, perceptions, and motivations of patients with HF enrolled in a structured telemedicine programme centered on wearable-based telemonitoring and multi-parameter remote monitoring, aiming to generate patient-centered insights to guide the design, governance, and scaling of telemedicine services for chronic HF.

## 2 Methods

### 2.1 Design

This qualitative study used a content analysis design and was embedded within a real-world telemedicine programme for chronic HF delivered by a specialist cardiology service at a tertiary University Hospital in Italy. Semi-structured interviews were conducted with purposively sampled patients to capture variation in age, sex, HF severity, and duration of telemedicine exposure. All interviews were audio-recorded, transcribed verbatim, and anonymized. Data collection and analysis proceeded in parallel, following an inductive approach to identify meaning units, codes, categories, and overarching themes. Recruitment continued until data saturation was reached, defined as the point when additional interviews no longer yielded new categories or insights.

### 2.2 Eligibility criteria

Patients were eligible if they were >18 years, had a diagnosis of chronic HF according to current guidelines (6, 28), were able to provide written informed consent, had no severe hearing or visual impairment, and had access to a telephone or internet-enabled device (personally or via a caregiver) for participation in the telemedicine program. Exclusion criteria included alcohol or substance use disorder interfering with participation, concurrent enrollment in another clinical study potentially affecting the intervention or data collection, or unstable severe psychiatric disorder. Participants were recruited through consecutive sampling, and study reporting adhered to COREQ guidelines (29).

### 2.3 Ethical considerations

The study was approved by the Ethics Committee of Sardinia (protocol no. 448/2025) and conducted in accordance with the Declaration of Helsinki (30). Data collection and storage complied with the General Data Protection Regulation. Potential participants were approached by a researcher and provided voluntary informed consent. Personal information was coded to ensure anonymity.

### 2.4 Procedures

The feasibility of the telemedicine intervention was first confirmed, ensuring adequate staff training, sufficient financial resources, and environmental sustainability. Patient enrollment occurred in two phases: clinical and technical. In the clinical phase, clinicians explained the telemonitoring programme and its objectives, emphasizing that it was not an emergency service. After obtaining informed consent, patients’ medical histories and current therapies were collected and uploaded to the telemonitoring platform.

In the technical phase, patients were trained by a technician to use wearable and home monitoring devices and the telemedicine application. Devices were installed, paired with the platform, and patients were taught to perform measurements and enter data using a teach-back approach. Patients were asked to perform three daily measurements, with at least one measurement if the full schedule was impracticable. An instruction booklet and technical support (12 h/day) were provided. Alarm thresholds for heart rate, oxygen saturation, blood pressure, and body weight were defined according to HF and telemonitoring literature (6, 28, 31–34), generating automated yellow or red alerts for deviations.

Each patient received a CE-marked smartwatch capable of automatically measuring HR, BP, and SpO_2_ every 15 min, as well as recording physical activity and sleep. Additional devices included an automatic sphygmomanometer, a ring pulse oximeter, and a single-lead ECG band. Data and alerts were transmitted to a dedicated telemedicine platform, enabling bidirectional exchange with healthcare professionals.

Nurses initially reviewed alerts for reliability and clinical significance, escalating valid alerts to physicians. Red alerts were prioritized. Physicians contacted patients via video call or telephone to assess anomalies and determine appropriate actions (e.g., medication adjustment, urgent visit, repeat measurements, or lab tests). Response times varied by alert severity: red alerts were addressed within ∼24 working hours, with faster intervention for multiple simultaneous alerts, while yellow alerts triggered monitoring based on the number of abnormal parameters, potentially prompting follow-up or treatment adjustment after 7 days. Non-working days were excluded from response-time calculations.

The programme supplemented, but did not replace, conventional care; patients were instructed to contact emergency services if needed.

### 2.5 Data collection

Data were collected from June to October 2025. After a three-month telemonitoring period, semi-structured face-to-face interviews were conducted in a private cardiology ward room, with only the participant and researcher present. Two trained researchers (one Ph.D., one master’s in nursing sciences), with no prior relationship to participants, facilitated interviews in a permissive, non-judgmental manner, allowing participants to speak freely. All interviews were audio-recorded, transcribed verbatim, and checked for accuracy. Following Kallio et al. (35), a semi-structured interview guide with 10 open-ended questions was developed and iteratively refined by a multidisciplinary team (Table 1), covering: initial impressions of the telemedicine journey; relationships with healthcare professionals; interaction with the technology; perceived changes in living with HF; perceived benefits and downsides; feelings about remote monitoring of medical data; family perspectives; and suggestions for improvement. Each participant was interviewed once, with field notes taken during and after the session. Interviews lasted approximately 40 min on average.

**TABLE 1:** Interview guide on patients’ experience with telemedicine.

Categories	Questions
Introductory question	How was it starting this telemedicine journey?
Transition questions	How was the relationship with telemedicine healthcare professionals?
Transition questions	How was your interaction with this new technology?
Key questions	Is there anything that seems to have changed in the way you experience the disease during telemedicine?
Key questions	Is there any way you think telemedicine has helped you?
Key questions	How does it feel to know that your medical data was being monitored remotely?
Key questions	Were there any downsides to telemedicine?
Key questions	What did your family think about telemedicine?
Key questions	Is there anything that could be improved in telemedicine?
Ending question	If you were to tell this telemedicine experience to someone who’s just starting out, what would you say?

### 2.6 Data analysis

Interview transcripts were analyzed using inductive content analysis following Graneheim and Lundman (34). Each transcript was read multiple times to ensure accurate understanding, and salient words and expressions were extracted as meaning units. Similar meaning units were assigned the same codes, which were then grouped into subcategories and broader categories based on similarities and differences. Analysis was conducted by two trained researchers under the supervision of two qualitative research experts. Two researchers independently coded the transcripts and met regularly to compare codes and category development. Coding disagreements were resolved through discussion until consensus; when consensus was not immediately reached, the issue was reviewed with the senior qualitative researchers, and decisions were documented in a shared coding log. The coding framework was iteratively refined as analysis progressed across interviews.

### 2.7 Rigor

To ensure rigor, we adhered to Lincoln and Guba’s (36) criteria of credibility, transferability, dependability, and confirmability. Credibility was maintained through an appropriate data collection strategy and sufficient data volume. Reflexivity focused on potential influences of the researchers’ clinical and academic backgrounds on coding and categorization, rather than on developing latent or explanatory interpretations; these issues were discussed within the multidisciplinary team and documented in the audit trail. Deviant or contrasting accounts were considered during category refinement to support confirmability. Transcripts were not returned to participants for member checking to minimize participant burden. Credibility was supported through verbatim quotations, field notes, and iterative peer debriefing of codes and categories within the research team.

Transferability was supported by detailed descriptions of the cultural and contextual setting, patient characteristics, data collection, and analysis procedures. Dependability was ensured by a clearly documented and logically structured research process. Confirmability was achieved by demonstrating consistent alignment between the data and the resulting interpretations, findings, and conclusions.

### 2.8 Sample

Patients learned about the study involving telemedicine through advertisements on signs inside the cardiology unit, as well as through conversations with cardiologists and nurses in the unit. Patients at the end of the telemedicine session were approached and asked if they were willing to be interviewed regarding their experiences with telemedicine. Twenty-one interviews achieved data saturation.

## 3 Results

### 3.1 Participants’ characteristics

**FIGURE 1:**
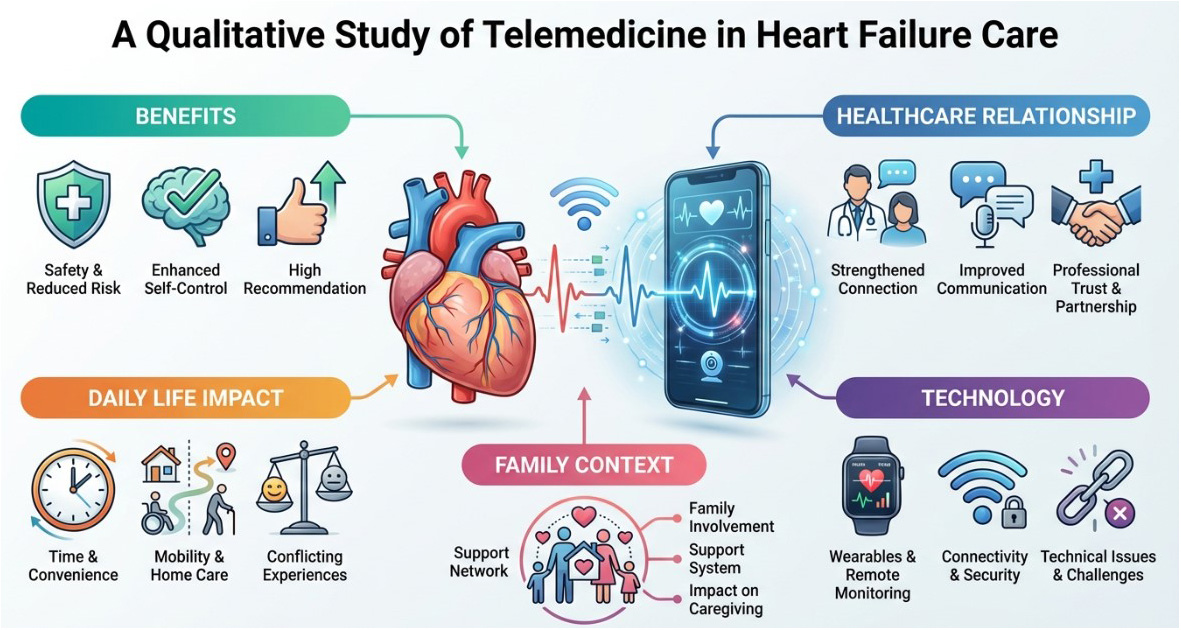
Infographic summarizing key findings from a qualitative study on telemedicine in heart failure care, organized into benefits, daily life impact, healthcare relationship, family context, and technology, using icons and brief phrases under each category for clarity.

The mean age of participants was 63 years, with most being men (76.2%) and Italian (95.2%). A majority had completed secondary education (71.4%). Common cardiovascular risk factors included dyslipidaemia (85.7%) and hypertension (66.6%), while 42.8% were overweight, 19% had type 2 diabetes, and 9.5% were current smokers. One third had atrial fibrillation (33.5%), and 47.6% had non-ischemic heart disease. HF phenotypes were distributed as reduced ejection fraction (33.4%), mildly reduced (28.6%), and preserved (38.0%). Over half had an implantable cardioverter-defibrillator (52.4%), 4.8% had chronic kidney disease, and 38.0% had chronic pulmonary disease. Patients took an average of 8.1 (SD 3.4) medications. Participant characteristics are summarized in Table 2, providing context for interpreting the qualitative findings. Figure 1 provides a graphical summary of the main study results.

**TABLE 2:** Participant characteristics.

Characteristics	Classification	*n*	%
Sex	Women	5	23.8
	Men	16	76.2
Age (years)	Mean (SD)	63 (10)	
Nationality	Italian	20	95.2
	Swiss	1	4.8
Education level	Primary school	2	9.5
	Secondary school	15	71.4
	High school	4	19.1
Arterial hypertension	Yes	14	66.7
	No	7	33.4
Dyslipidaemia	Yes	18	85.7
	No	3	14.3
Overweight	Yes	9	42.9
	No	12	57.2
Type II diabetes mellitus	Yes	4	19
	No	17	81
Smoker	Yes	2	9.5
	No	19	90.5
Atrial fibrillation	Yes	7	33.3
	No	14	66.5
Non-ischemic heart disease	Yes	10	47.6
	No	11	52.4
Heart failure with reduced ejection fraction (HFrEF)	Yes	7	33.4
	No	14	66.6
Heart failure with mildly reduced ejection fraction (HFmrEF)	Yes	6	28.6
	No	15	71.4
Heart failure with preserved ejection fraction (HFpEF)	Yes	8	38
	No	13	62
Ejection fraction	Mean (SD)	44.09	12.8
Pacemaker	No	21	100
ICD	Yes	11	52.4
	No	10	47.6
CRT	Yes	1	4.8
	No	20	95.2
Chronic renal failure	Yes	3	14.3
	No	18	85.7
Pulmonary diseases	Yes	8	38
	No	13	62
Medications	Mean (SD)	8.09	3.4

SD, standard deviation; ICD, implantable cardioverter-defibrillator; CRT, cardiac resynchronization therapy.

### 3.2 Findings

Data saturation was reached during qualitative data collection. Analysis identified 13 subcategories grouped into five main categories: (1) benefits of telemedicine, (2) impact on daily life, (3) relationship with healthcare professionals, (4) interaction with technology, and (5) family context.

Table 3 provides an overview of the analytic structure linking categories, subcategories, and condensed meaning units. Below, we describe each category and provide illustrative quotations to show how these themes were expressed in participants’ accounts. Participants are identified by alphanumeric codes in parentheses after each quote.

**TABLE 3:** Findings of qualitative analysis.

Main categories	Subcategories	Codes	Condensed meaning units
Benefits of telemedicine	Perceived safety and reassurance	Reassurance	Participants reported feeling safe
	–	Monitoring	Participants perceived continuous clinical monitoring
	Greater self-control	Awareness	Participants reported increased awareness of their clinical status
	Recommendation of telemedicine	Usefulness	Participants would recommend telemedicine to others
Impact on daily life	Time impact	Commitment	Participants described telemonitoring as requiring ongoing commitment
	–	Deadlines	Participants experienced fixed deadlines associated with the program
	Practical limitations and mobility	Constraints	Participants felt constrained in their movements and daily planning
	Conflicting experience	Ambivalence	Participants expressed ambivalent feelings toward telemedicine
Relationship with healthcare professionals	Presence of healthcare professionals	Proactivity	Participants felt actively followed up by healthcare professionals
	Desire for greater contact	Feedback	Participants perceived insufficient or delayed feedback from professionals
	Professional behaviors	Professionalism	Participants recognized a high level of professionalism in the staff
Interaction with technology	Unreliable devices	Hardware and software problems	Participants reported some hardware and software malfunctions
	Unreliable connectivity	Connectivity problems	Participants occasionally had issues with unstable or dropped connections
Family context	Family support	Help	Participants received practical support from family members
	–	Serenity	Participants perceived reassurance and calmness among family members
	Family members’ attitudes	Worry	Participants perceived relatives’ concern regarding their health condition
	–	Curiosity	Participants noticed relatives’ curiosity about telemedicine and devices
	–	Control	Participants perceived relatives’ tendency to control or supervise telemedicine devices

#### 3.2.1 Benefits of telemedicine

This category comprised three subcategories: *perceived safety and reassurance*, *greater self-control*, and *recommendation of telemedicine*, reflecting patients’ perceived benefits.

Development of clinical safety: Participants reported feeling safer and continuously monitored, perceiving telemedicine as a protective mechanism that could detect early deterioration (e.g., “It gives me security, in the sense that I know there’s someone monitoring me…” [ID 1]; “I feel monitored…” [ID 9]).

Greater self-control: Access to clinical information enhanced patients’ awareness of their condition and promoted more frequent self-monitoring of parameters such as blood pressure and heart rate (e.g., “I check myself a few more times…” [ID 5]; “I was able to monitor my blood pressure during the period…” [ID 15]).

Recommendation of telemedicine: Most participants considered the program useful and would recommend it to others, highlighting its feasibility and minimal interference with daily life (e.g., “I would strongly recommend it to you, without any doubts…” [ID 3]; “I would recommend it to you, because it doesn’t bother me at all…” [ID 8]).

#### 3.2.2 Impact on daily life

This category comprised three subcategories: *time impact*, *practical limitations and mobility*, and *conflicting experience*, reflecting patients’ perceptions of how telemedicine affected daily routines.

Time impact: Telemedicine was experienced as an additional time commitment, structured by fixed schedules and repeated measurements, which could feel demanding or constrain daily activities (e.g., “It’s a bit demanding, even following the timetables…” [ID 3]; “At the beginning it was a bit heavy because of the timetables…” [ID 7]).

Practical limitations and mobility: Some participants felt anchored to technology, experiencing constraints on mobility due to device use or connectivity requirements (e.g., “The bad thing is that you can’t go out because the Wi-Fi is down…” [ID 1]; “I have to try to get everything done in time to get back, to take the various parameters…” [ID 3]).

Conflicting experience: Patients described ambivalent feelings, recognizing both benefits and burdens of telemedicine, including perceptions of control and potential over-monitoring (e.g., “It’s a fantastic technology because it helps, but when those limits are exceeded, it’s harmful…” [ID 9]; “Until now, I thought that remotely monitored data was only for study and research purposes… now it’s actually a form of control…” [ID 5]).

#### 3.2.3 Relationship with healthcare professionals

This category included three subcategories: *presence of healthcare professionals*, *desire for greater contact*, and *professional behaviors*, reflecting patients’ perceptions of their interactions with clinicians in the telemedicine context.

Presence of healthcare professionals: Participants felt supported by proactive healthcare staff who monitored their condition and maintained contact (e.g., “They told me they were following me…” [ID 5]; “I feel more at ease because I am being supported…” [ID 15]).

Desire for greater contact: Some patients expressed the need for more frequent or structured feedback, beyond occasional checks (e.g., “Only two minutes of contact, every now and then…” [ID 5]; “No one ever contacted us, perhaps because everything was going well…” [ID 12]).

Professional behaviors: Patients valued professionalism, clarity, and empathy in clinician interactions, which fostered trust and confidence (e.g., “I see serious, attentive people… there’s a relationship of trust…” [ID 9]; “They explained everything to me…” [ID 13]).

#### 3.2.4 Interaction with technology

This category included two subcategories: *unreliable devices* and *unreliable connectivity*, reflecting patients’ experiences with the telemedicine system’s hardware, software, and network components.

Unreliable devices: Participants reported discomfort and functional limitations with devices and applications, including issues with blood pressure cuffs, wearable rings and watches, battery life, and occasional app malfunctions or freezes (e.g., “The blood pressure cuff sometimes didn’t work properly…” [ID 15]; “The watch’s battery life is a bit short…” [ID 1]; “The app would freeze and required frequent updates…” [ID 10]).

Unreliable connectivity: Patients experienced connectivity problems that interfered with measurements and data transmission (e.g., “The connections that sometimes drop are very annoying…” [ID 5]; “It’s a connection-related issue…” [ID 13]).

#### 3.2.5 Family context

This category included two subcategories: *family support* and *family members’ attitudes*, reflecting patients’ experiences with family involvement during telemedicine.

Family support: Participants reported practical and emotional support from family members in using telemedicine, which also provided reassurance to relatives (e.g., “Both my daughter and my wife helped me a little…” [ID 5]; “I couldn’t understand and do it well, and they were happy to help me…” [ID 7]; “They are also more relaxed…” [ID 2]).

Family members’ attitudes: Patients noted relatives’ curiosity, concern, and sometimes controlling behaviors regarding telemedicine devices (e.g., “They see that it’s a bit difficult, because it’s a new thing anyway…” [ID 14]; “They’re curious about the bathroom scales… they’ve put themselves on it too…” [ID 1]; “When we talk to my daughter who’s a pharmacist I’m also checked on a family level…” [ID 4]).

## 4 Discussion

This qualitative study explored the experiences of HF patients participating in a structured telemedicine programme (37). Overall, telemedicine was framed positively by most participants, primarily in terms of reassurance, perceived clinical safety, and enhanced self-monitoring and self-control. At the same time, ambivalence emerged as a recurrent, structurally embedded feature of the experience: the same “always-on” monitoring and alert-driven routines that generated reassurance also produced burden (time demands, rigid measurement schedules), technological dependence, and occasional feelings of being monitored or controlled. Taken together, participants’ accounts suggest that perceived benefits generally outweighed perceived burdens, but acceptability remained contingent on maintaining a favorable benefit–burden balance. In a wearable-enabled, multi-parameter telemonitoring model with predefined alerts and a structured response pathway, the themes should be interpreted in relation to continuous monitoring, measurement routines, and clinician follow-up. Overall, telemedicine was perceived as beneficial for clinical safety, self-monitoring, and acceptability, consistent with a recent qualitative meta-synthesis in cardiovascular digital health highlighting reassurance, emotional support, and empowerment. Importantly, our findings extend prior qualitative work by showing how these experiences unfold within an alert-driven telemonitoring service integrated into routine HF care, generating an “always-on” sense of being watched over and new expectations regarding responsiveness and accountability.

Participants frequently described telemedicine as increasing reassurance and perceived safety through continuous monitoring and timely clinical review, echoing prior qualitative evidence on feeling “continuously accompanied” (38–42) and studies suggesting that monitoring combined with clinician oversight can strengthen engagement in self-care (43, 44).

Participants reported greater awareness of their clinical status and more frequent self-monitoring of blood pressure, weight, heart rate and other parameters, consistent with evidence that structured tracking and feedback can strengthen confidence and perceived control (45, 46) and with findings from hypertensive cohorts using similar platforms (37). Overall acceptability was high: most participants considered telemedicine useful and would recommend it, in line with prior work on cardiac apps and eHealth rehabilitation (44, 46, 47). However, acceptability depended on a favorable benefit–burden balance. Fixed schedules, frequent measurements, and device-related routines were experienced as time-demanding and sometimes as “deadlines” that structured the day and could generate pressure, echoing reports of telemonitoring competing with work and family responsibilities (38, 48). These findings suggest that monitoring intensity and workflow expectations shape this balance; flexible measurement windows, low-burden devices, and tailoring monitoring frequency to clinical risk and patient preferences may reduce perceived burden while maintaining clinical benefit.

Several participants reported feeling “tied to the technology,” unable to stay away from home or travel freely due to reliance on Wi-Fi or proximity to devices. Similar constraints have been observed in cardiac rehabilitation and remote monitoring programs (48). Even with good connectivity, telemedicine can anchor patients to specific times and places; improving device mobility/interoperability and designing workflows that accept occasional missed or delayed measurements may reduce this burden.

Telemedicine was often described as a “fantastic technology” but also as potentially “harmful” when its limits were exceeded or when patients felt more “controlled” than supported. This ambivalence reflects previous qualitative research among individuals with low acceptance of HF mobile health apps, who similarly voiced doubts about utility, concerns about dependence, and uncertainty regarding clinicians’ actual use of transmitted data (49). These findings highlight the need to address the “double nature” of telemedicine during patient education, clarifying benefits and limitations and establishing shared expectations about data use, visibility, and responsiveness.

The relationship with healthcare professionals was central in shaping patient experience. Participants valued feeling monitored and supported, and the perception that clinicians were “continuously following” them, reinforced by periodic calls, enhanced reassurance. This is consistent with evidence from HF and hypertension digital interventions showing that patients appreciate active review of transmitted data and clinician-initiated contact when concerning trends appear (50, 51). As noted in Sivakumar et al., perceived usefulness depends on clinician oversight rather than self-tracking alone (43). Our findings suggest that telemedicine strengthens continuity of care when patients believe their data meaningfully inform clinical decisions.

Despite feeling supported, some participants desired more regular or scheduled feedback rather than contact only when “something was wrong.” Similar expectations have been described among HF app users who experienced disappointment when digital tools offered one-way communication or untimely responses (46). This expectation may reflect how structured alert management and response workflows are experienced by patients, highlighting the value of making response processes and anticipated timelines explicit during onboarding. This supports the need for flexible telemedicine models that include proactive, relational contact for those who prefer more continuous engagement.

Participants described healthcare professionals as serious, attentive, kind, and clear in explaining both technological and clinical aspects. This aligns with qualitative evidence from nurse-led eHealth and smartphone-based programs, where structured onboarding, empathic communication, and step-by-step guidance were essential for confidence and adherence (47, 52), and with findings that trust in eHealth interventions is strongly influenced by clinicians’ attitudes and personalized guidance (53). Our results emphasize that the human relationship remains pivotal despite digital mediation and that clinicians require training in both technical and relational digital competencies.

Technical factors strongly shaped patient experience. Participants reported issues such as limited smartwatch battery life, uncomfortable straps, and the need to keep smartphones constantly charged. These concerns are consistent with studies showing that discomfort, cumbersome multi-device setups, and hardware complexity decrease usability and satisfaction (45, 48, 54). Intermittent Wi-Fi and connectivity failures, which sometimes prevented measurements or data transmission, were also problematic, echoing reports from diverse settings (49, 51). These findings underscore the importance of robust, ergonomic devices with minimal maintenance demands and of redundancy strategies to mitigate connectivity disruptions.

Family context was another key facilitator of engagement, offering both practical assistance and emotional reassurance. Many participants reported that relatives helped them use devices, interpret instructions, and integrate telemonitoring into daily routines, while family members felt more relaxed knowing monitoring was ongoing. Similar patterns of family involvement have been observed in East Asian cardiac rehabilitation and mHealth studies (52, 54), where caregiver access to app data is often explicitly requested (37).

Some caregivers were perceived as overly “controlling,” using devices themselves or closely monitoring adherence. This dual role, supportive yet supervisory, aligns with prior research showing that family involvement can both empower and pressure patients (55). Telemedicine programs should explicitly define family roles, data-access rights, and boundaries to promote supportive engagement without intrusion. Across themes, the central contribution of this study lies in the ambivalences that patients negotiated within a high-intensity, wearable-enabled monitoring model. The same features that generated reassurance (continuous data capture and clinician oversight) also produced feelings of constraint (measurement routines, device dependence, and reduced mobility), while clinician-initiated contact following alerts strengthened trust but sometimes reinforced a preference for more proactive, scheduled feedback. These tensions suggest that acceptability is not a fixed attribute of “telemedicine” *per se*, but is shaped by how monitoring intensity, alert management, and communication practices are implemented in everyday life.

Overall, our findings, integrated with recent meta-synthesis evidence in cardiovascular digital health (37), suggest that telemedicine experiences are shaped by tensions between reassurance and burden, autonomy and dependence, individual and family involvement, and human and technological presence. Anticipating and addressing these dynamics is crucial for designing clinically effective, patient-centered telemedicine models.

This study has some limitations. It represents a single-center experience within a specific telemedicine program, limiting generalizability. Only patients who completed the program and met eligibility criteria were included, likely under-representing individuals with sensory impairments, lower education, or limited health literacy; this may have limited the representation of experiences related to technological burden or access barriers and may have contributed to a more favorable overall picture of acceptability, with implications for digital equity. The sample was also relatively homogeneous and excluded patients with significant sensory or cognitive limitations, which may have limited insights into equity-related barriers. In addition, transcripts of the audio-recorded interviews were not returned to participants for member checking to minimize participant burden, which may have limited opportunities to verify the researchers’ understanding of participants’ accounts; credibility was supported through field notes, verbatim quotations, and team-based peer debriefing. No comparative analyses across age or sociodemographic subgroups were conducted, preventing exploration of potential differences related to age, sex, digital literacy, or family context. Future research should include larger, more diverse samples with subgroup comparisons to better understand variations in telemedicine experiences, including potential differences related to HF phenotype (HFrEF/HFmrEF/HFpEF) (28), which may shape patients’ perceptions, routines, and expectations of telemonitoring due to phenotype-related heterogeneity in symptom expression and comorbidity burden: for example, HFpEF is often characterized by older age, multimorbidity/frailty and more non-specific or fluctuating symptom patterns with competing symptom drivers (cardiac vs. extracardiac), which may complicate symptom perception and weaken the perceived link between HF-specific behaviors and day-to-day benefits; in contrast, HFrEF/HFmrEF may follow a clearer HF-specific therapeutic trajectory with more readily interpretable symptom–self-care contingencies and more defined monitoring targets (56, 57).

## 5 Conclusion

Telemedicine can improve perceived safety and reassurance, self-management, continuity of care, and treatment adherence in HF patients when embedded within strong patient–clinician relationships, supported by reliable technology, and integrated into daily routines and family dynamics. Future programs should be co-designed with patients and caregivers, provide flexible personalized monitoring, clarify data use, and ensure effective two-way communication. Multicenter, longitudinal mixed-methods studies are warranted to examine how organizational, technological, and socio-cultural factors shape patient experiences and to identify telemedicine models that optimally support person-centered HF care.

## Data Availability

The raw data supporting the conclusions of this article will be made available by the authors, without undue reservation.
